# Bacteriophage PCSE1 as a Potential Strategy Against *Salmonella* Enteritidis in Liquid Egg Products

**DOI:** 10.3390/antibiotics14080811

**Published:** 2025-08-08

**Authors:** Márcia Braz, Carla Pereira, Gabriela Matos, Jorge A. Saraiva, Carmen S. R. Freire, Adelaide Almeida

**Affiliations:** 1 Centre for Environmental and Marine Studies (CESAM), Department of Biology, University of Aveiro, Campus Universitário de Santiago, 3810-193 Aveiro, Portugal; marciabraz96@ua.pt (M.B.); csgp@ua.pt (C.P.); 2 CICECO-Aveiro Institute of Materials, Department of Chemistry, University of Aveiro, Campus Universitário de Santiago, 3810-193 Aveiro, Portugal; cfreire@ua.pt; 3 ALS Life Sciences Portugal, S.A, Zona Industrial De Tondela Lote 2-6 Adiça, Lt 6 Adiça Mouraz Tondela, 3460-070 Tondela, Portugal; 4 Associated Laboratory for Green Chemistry (LAQV) of the Network of Chemistry and Technology (REQUIMTE)—LAQV-REQUIMTE, Department of Chemistry, University of Aveiro, Campus Universitário de Santiago, 3810-193 Aveiro, Portugal; gabrielamatos@ua.pt (G.M.); jorgesaraiva@ua.pt (J.A.S.)

**Keywords:** *Salmonella* Enteritidis, egg contamination, bacteriophage, biocontrol, pasteurization, egg properties, food safety

## Abstract

Background/Objectives: The consumption of liquid egg products is rising. While thermal pasteurization improves safety and shelf life, it can affect product quality. Furthermore, egg products continue to cause many foodborne illnesses, especially those caused by *Salmonella enterica* subspecies *enterica* serovar Enteritidis (*Salmonella* Enteritidis). Bacteriophages (or phages) are an effective alternative to specifically fight foodborne bacteria. This study aimed to evaluate (i) the stability of phage vB_SeEM_UALMA_PCSE1 (PCSE1) under different conditions of temperature and pH; (ii) the effect of multiplicity of infection (MOI) and temperature on phage efficacy; (iii) the bactericidal effect of phage PCSE1 against *S.* Enteritidis in liquid whole eggs compared to thermal pasteurization; and (iv) the effect of both treatments on the physicochemical and functional properties of liquid whole eggs. Methods: For this, stability tests, bacterial growth inhibition assays in culture media and liquid eggs, and physicochemical and functional analyses were conducted. Results: Phage PCSE1 was (i) stable at pH 7 and 8, and at 4, 25, and 37 °C for 56 days; (ii) effectively prevented *S.* Enteritidis growth in TSB (reduction of 1.8, 4.5, and 4.5 log colony-forming units (CFU)/mL at 4, 10, and 25 °C, respectively, relative to the bacterial control); (iii) controlled *S.* Enteritidis in liquid whole eggs at 25 °C (reduction of 5.8 log CFU/mL relative to the bacterial control) comparable to pasteurization (reduction of 5.2 log CFU/mL); and (iv) preserved eggs’ properties, contrarily to pasteurization. Conclusions: These findings suggest PCSE1 is a promising strategy to fight *S.* Enteritidis in liquid egg products, though further studies on shelf-life are needed.

## 1. Introduction

Eggs have been a fundamental food source worldwide due to their high nutritional value, low cost, and functional properties such as emulsifying, gelling, and foaming, which contribute to their food texture and sensory qualities [1,2]. While shell eggs are preferred in many industries, those that do not meet the quality standards are processed into egg products, which offer practical advantages for large-scale manufacturing [1].

Liquid egg products retain the nutritional benefits of whole eggs and address the issues related to fragility, transportation and storage, and pathogen transmission [1,2]. European regulations define “egg products” as processed eggs intended for human consumption [3]. In some cases, such as large events, the use of egg products instead of raw eggs is mandatory [4]. Processing steps include breaking, filtering, mixing, stabilizing, blending, pasteurizing, cooling, freezing, drying, and packaging, though washing eggs is prohibited in some EU countries to avoid damaging the protective eggshell and cuticle, which help prevent bacterial contamination [1,5].

*Salmonella* is rarely found in pasteurized egg products, but can persist due to heat-resistant bacteria or post-processing contamination [6]. Eggs and egg products remain a major source of salmonellosis outbreaks, with *Salmonella enterica* serovar Enteritidis being the most common serovar linked to contaminated eggs worldwide [7,8,9]. Globally, non-typhoidal *Salmonella* causes approximately 150 million illnesses and 60,000 deaths annually [10], with symptoms including nausea, vomiting, diarrhea, and fever [11].

Thermal pasteurization is the standard method to improve the microbiological safety and shelf life of liquid eggs, with conditions that can vary by country [2]. However, heat treatment can damage heat-sensitive egg proteins, affecting quality by causing protein precipitation and reduced foaming ability [12,13,14]. Additionally, pasteurized products require refrigeration and have a limited shelf life of a few weeks [15]. This, together with the survival of some heat-resistant bacteria, including *S.* Enteritidis, highlights the need for alternative or complementary methods to enhance the safety, quality, and shelf life of liquid eggs [2,6].

Different non-thermal techniques, including pulsed electric field, ultraviolet radiation, and ultrasound, have been explored to control foodborne pathogens while preserving the nutritional and quality attributes of liquid eggs. However, these methods often require specialized equipment and technical expertise, increasing production costs [2]. Therefore, there is a growing interest in cost-effective alternatives, such as bacteriophages, as promising biocontrol agents [16].

Bacteriophages (phages) are naturally occurring viruses that specifically infect bacteria and are the most abundant biological entities on Earth [17]. Phages are promising natural biocontrol agents for application in food [18] due to their high specificity, safety, self-replication in the presence of target bacteria, without impacting food quality, and beneficial microbiota [16,19]. They have FDA’s Generally Recognized as Safe (GRAS) status and several commercial phage products targeting *Salmonella* are available, such as PhageGuard S^TM^, SalmoFresh™, and SalmoPro^®^, applied in different food matrices including meat, fish and shellfish, fruits, vegetables, and eggs [19,20,21,22].

Several studies have demonstrated the potential of phages against *S.* Enteritidis in eggshell [23,24,25,26,27] and separated liquid egg components such as egg whites and yolks [28,29,30,31]. However, to the best of our knowledge, to date, only four studies have investigated phage application against *S.* Enteritidis in liquid whole eggs [23,32,33,34]. Hong et al. (2016) [32] showed that phage SJ2 (MOI 10) significantly reduced *S.* Enteritidis levels by 0.6–0.9 log CFU/mL at 21 °C, though it was ineffective at 4 °C. Duc et al. (2020) [33] reported reductions of 1.0 and 2.0 log CFU/mL at 4 and 24 °C, respectively, using phage PS5 at a high MOI (10,000). Azari et al. (2023) [23] detected bacterial reductions below the detection limit at 4 °C with phage Rostam (MOI 10,000) and observed a reduction of ~4.5 log CFU/mL at 25 °C. Torkashvand et al. (2024) [34] found that phage E19 (MOI 100,000) completely eradicated *S.* Enteritidis in 30 min and suppressed regrowth for 72 h. These studies highlight the importance of both MOI and temperature in phage efficacy. While higher MOIs increase the likelihood of contact between the phage and host, the ability of phages to replicate in food, especially in liquid matrices [35,36], suggests that lower MOIs may also be effective. Temperature plays a key role, as lower temperatures reduce bacterial metabolism and, consequently, phage replication [33], potentially limiting their effectiveness.

Considering the reduced number of available studies on phage application in liquid whole eggs, and mainly using high MOIs, there is room for further investigation, given the association between egg consumption and *S.* Enteritidis infection [7,8].

Despite their promising features, several challenges still hinder the widespread use of phages in the food industry [21,37]. One major concern is bacterial resistance. Although phage-resistant strains often exhibit reduced pathogenicity [38], phages can evolve rapidly to overcome resistance. The use of phage cocktails, targeting multiple receptors, and in combination with other antimicrobials, can also help dealing with this issue [37]. Another challenge is phage specificity and narrow host ranges, which may limit effectiveness in food contexts with diverse strains—however, cocktails may also be a solution [39]. Additionally, phage stability and activity are influenced by environmental factors such as pH, temperature, food matrices, and the presence of antimicrobials [21,23]. While several studies have evaluated phage stability under different pH and temperature conditions, these are typically limited to short incubation times (1–2 h) [24,40,41,42]. Longer-term stability assessments are essential, particularly in the context of food storage.

Given the negative impact of thermal pasteurization on liquid whole egg quality and the growing demand for minimally processed foods [2], phages represent a promising alternative/complement to control *Salmonella* contamination and reduce salmonellosis risk. Although phages are generally reported to not alter food properties [43,44], studies evaluating their effects are scarce [45], particularly in liquid whole eggs, where, to the best of our knowledge, no studies on the evaluation of the physicochemical and functional properties are available.

Therefore, this study aimed to assess, for the first time, the potential of phage PCSE1, previously isolated and characterized by our group [46], as a biocontrol agent against *S.* Enteritidis in liquid whole eggs. Specifically, we evaluated the following: (i) phage PCSE1 stability at different temperatures (4, 25, 37, and 45 °C) and pH values (3, 5, 7, and 8) over long storage; (ii) the effect of MOI (1, 10, 100, and 1000) and temperature (4, 10, and 25 °C) on phage efficacy in Tryptic Soy Broth (TSB); (iii) the bactericidal effect of phage PCSE1 in liquid whole eggs at 25 °C, using the best conditions from TSB assays; (iv) phage effectiveness compared to conventional thermal pasteurization; and (v) the impact of phage treatment on key egg quality parameters (pH, color, soluble protein, viscosity, and foaming), relative to pasteurization.

## 2. Results

### 2.1. Phage Stability Under Different Temperature and pH Conditions

**Figure 1:**
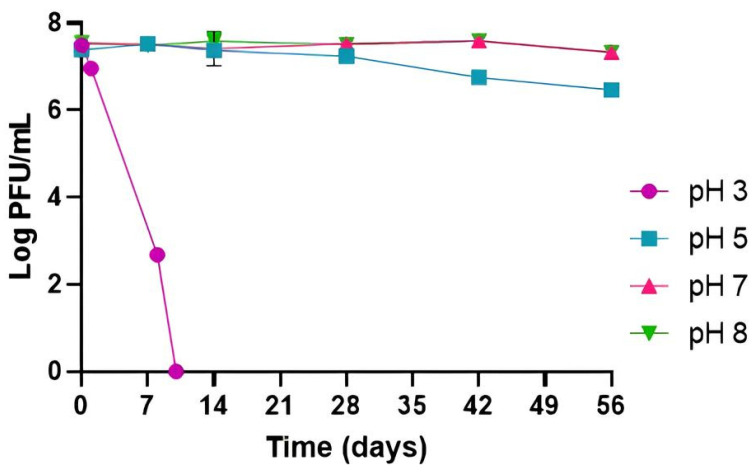
Phage PCSE1 stability at different pH values (constant temperature of 25 °C). Values represent the mean of three independent tests with error bars representing the standard deviation.

At pH 7 and 8, phage titer remained stable during the 56 days of the experiment, and without significant differences between them (*p* > 0.05). At a lower pH of 5, a decrease in phage titer of 0.9 log PFU/mL (*p* < 0.05) was obtained only at the end of the incubation period of 56 days (Figure 1). The highest decrease in phage titer was observed at pH 3, with a decrease of 4.8 log PFU/mL after 8 days, with 2.7 log PFU/mL remaining. A reduction to the detection limit of the method was obtained after 10 days (*p* < 0.05).

**Figure 2:**
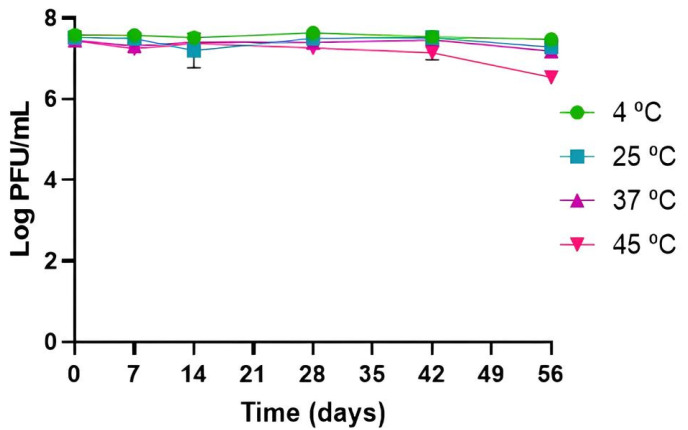
Phage PCSE1 stability at different temperatures (constant pH of 7). Values represent the mean of three independent tests with error bars representing the standard deviation.

Phage PCSE1 was considerably stable under all tested temperatures, with phage titer remaining similar to the initial phage titer after 56 days (*p* > 0.05) at 4, 25, and 37 °C, without significant differences between them. A small decrease of 0.9 log PFU/mL was only observed at 45 °C (*p* < 0.05), after 56 days (Figure 2).

### 2.2. Bacterial Kill Curves with Phage Treatment in TSB

**Figure 3:**
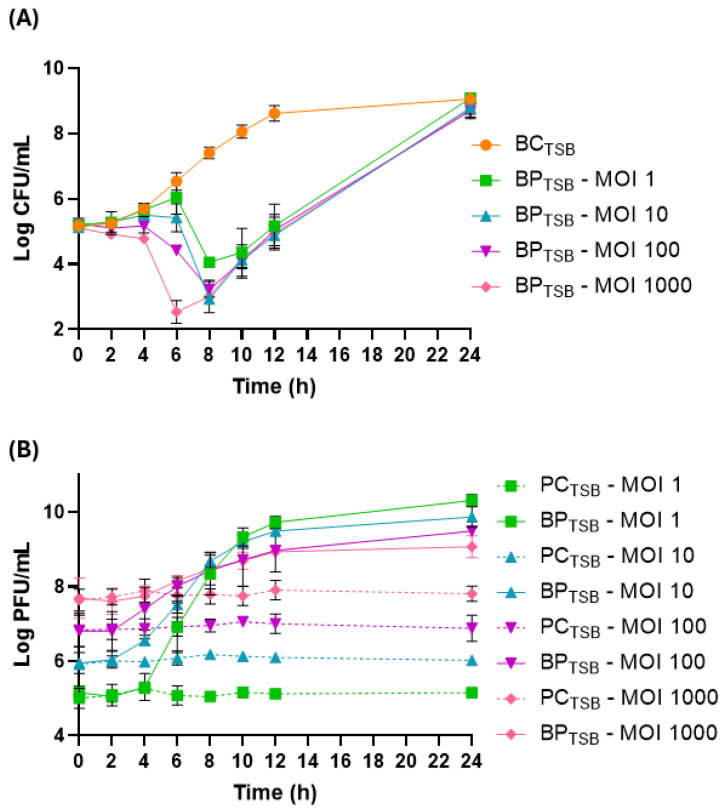
Reduction in *S.* Enteritidis levels by phage PCSE1 at an MOI of 1, 10, 100, and 1000 during 24 h at 25 °C in TSB. (**A**) Bacterial concentration: BC_TSB_—*S.* Enteritidis control with only bacterium; BP_TSB_—*S.* Enteritidis plus phage PCSE1. (**B**) Phage concentration: PC_TSB_—phage PCSE1 control with only phage; BP_TSB_—*S.* Enteritidis plus phage PCSE1. Data represent the mean of three independent tests with error bars representing the standard deviation.

At 25 °C, the bacterial density in the bacterial control (BC_TSB_) increased by 3.9 log CFU/mL (*p* < 0.05, Figure 3A) during the 24 h of incubation. Phage PCSE1 effectively prevented *S.* Enteritidis growth in the sample (BP_TSB_), compared to BC_TSB_, at a maximum of 3.7, 4.5, 4.2, and 4.4 log CFU/mL at MOI 1, 10, 100, and 1000, respectively, after 8 (MOI 10, 100, and 1000) and 10 h (MOI 1) (*p* < 0.05) (Figure 3A). Although a similar maximum difference was obtained for MOIs of 10, 100, and 1000, relative to each correspondent control, it was observed earlier for MOI 1000 (4.0 log CFU/mL after 6 h) compared to other MOI values (0.5, 1.1, and 2.1 log CFU/mL at MOI 1, 10, and 100, respectively). Significant differences (*p* < 0.05) between MOI values were only observed after 4 h (MOI 1/MOI 1000 and MOI 10/MOI 1000), 6 h (all MOIs except MOI 1/MOI 10), and 8 h (MOI 1/MOI 10, MOI 1/MOI 100, and MOI 1/1000). After the major decrease, bacterial regrowth was observed for all MOIs, with similar bacterial numbers observed in BC_TSB_ and BP_TSB_ after 24 h (*p* > 0.05).

All phage controls (PC_TSB_) remained constant throughout the assay (Figure 3B). In the sample (BP_TSB_), a significant increase (*p* < 0.05) in phage titer was observed for all tested MOIs, with a higher increase observed for lower MOIs (5.2, 3.8, 2.6, and 1.3 PFU/mL increase for MOI 1, 10, 100, and 1000, respectively), relative to each corresponding PC_TSB_.

**Figure 4:**
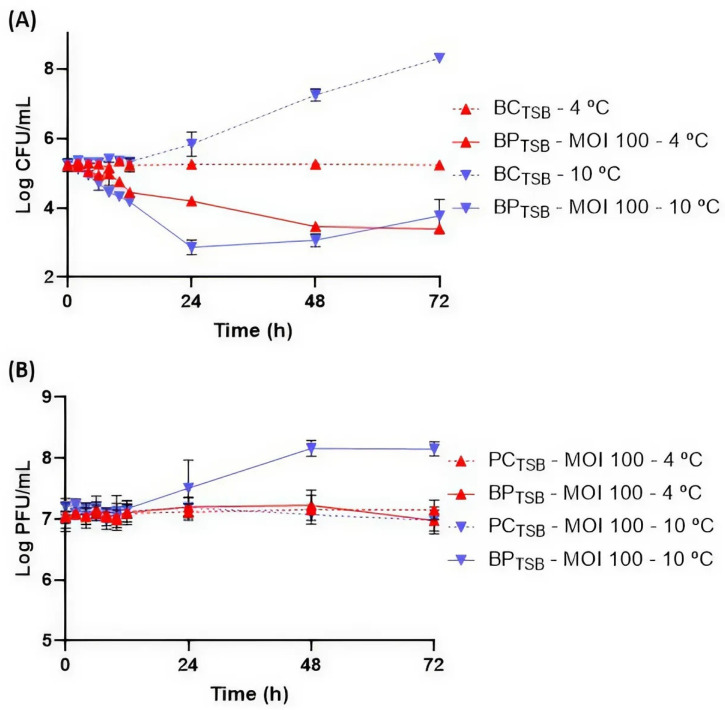
Reduction in *S.* Enteritidis levels by phage PCSE1 at an MOI of 100 during 72 h at 4 and 10 °C in TSB. (**A**) Bacterial concentration: BC_TSB_—*S.* Enteritidis control with only bacterium; BP_TSB_—*S.* Enteritidis plus phage PCSE1. (**B**) Phage concentration: PC_TSB_—phage PCSE1 control with only phage; BP_TSB_—*S.* Enteritidis plus phage PCSE1. Data represent the mean of three independent experiments with error bars representing the standard deviation.

At 10 °C, bacterial numbers in BC_TSB_ increased by 3.9 log CFU/mL (Figure 4A). In the presence of phage PCSE1, a maximum bacterial difference of 4.5 log CFU/mL was observed at the end of the treatment, after 72 h, in BP_TSB_ compared to BC_TSB_ (*p* < 0.05). A significant decrease of 2.9 and 4.1 log CFU/mL (*p* < 0.05) was already observed after 24 and 48 h, respectively. However, bacterial regrowth was observed after 24 h.

At 4 °C, BC_TSB_ remained stable throughout the assay (Figure 4A). A significant bacterial difference was observed after 10 h (0.5 log CFU/mL) in BP_TSB_ compared to BC_TSB_ (*p* < 0.05). A maximum difference was observed at the end of the treatment after 72 h (1.8 log CFU/mL). No bacterial regrowth was observed.

Regarding phage behavior, PC_TSB_ remained stable at 10 and 4 °C. An increase in phage PCSE1 titer in BP_TSB_ (1.2 log PFU/mL) was observed at 10 °C (*p* < 0.05), while at 4 °C, phage titer remained stable (*p* > 0.05) during the 72 h (Figure 4B).

### 2.3. Bacterial Kill Curves in Liquid Whole Eggs

#### 2.3.1. Phage Treatment

**Figure 5:**
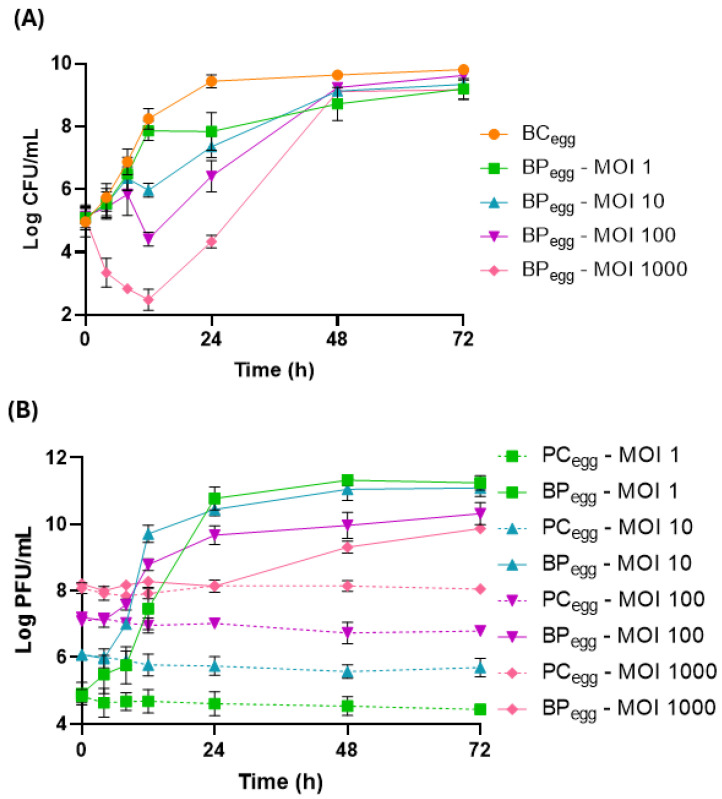
Reduction in *S.* Enteritidis levels by phage PCSE1 at an MOI of 1, 10, 100, and 1000 during 72 h at 25 °C in liquid whole eggs. (**A**) Bacterial concentration: BC_egg_—*S.* Enteritidis control with only bacterium; BP_egg_—*S.* Enteritidis plus phage PCSE1. (**B**) Phage concentration: PC_egg_—phage PCSE1 control with only phage; BP_egg_—*S.* Enteritidis plus phage PCSE1. Data represent the mean of three independent tests with error bars representing the standard deviation.

A maximum increase in *S.* Enteritidis concentration of 4.8 log CFU/mL was observed in the bacterial control (BC_egg_) after 72 h of incubation at 25 °C (Figure 5A). Phage PCSE1 effectively reduced bacterial counts in the sample (BP_egg_) in an MOI-dependent manner, when compared to BC_egg_ (*p* < 0.05), with higher differences for higher MOI values, namely, MOI 1000 (Figure 5A). Maximum bacterial differences in the sample, relative to the bacterial control, of 1.6, 2.3, 3.8, and 5.8 log CFU/mL were obtained for MOI 1, 10, 100, and 1000, respectively, after 12 (MOI 10, 100, and 1000) and 24 h (MOI 1). MOI 1000 was significantly different (*p* < 0.05) from the other MOIs during all the assays, except after 48 and 72 h, where there were no significant differences between any of the MOIs. Significant differences (*p* < 0.05) were also observed for other MOIs after 8 h (MOI 1/MOI 10, MOI 1/MOI 100, and MOI 10/MOI 100) and 12 h (MOI 1/MOI 100 and MOI 10/MOI 100). Even with bacterial regrowth, a significant difference between BP_egg_ and BC_egg_ was still observed after 24 h (*p* < 0.05) for all values of MOI. No bacteria were detected in the liquid whole egg control (not artificially contaminated and untreated).

Phage titer remained constant in all phage controls (PC_egg_) (*p* > 0.05, Figure 5B). An increase in phage titer was observed in the sample (BP_egg_) relative to each corresponding PC_egg_ (*p* < 0.05), with a higher increase for lower MOI values (6.8, 5.4, 3.5, and 1.8 log PFU/mL increase for MOI 1, 10, 100, and 1000, respectively).

#### 2.3.2. Thermal Pasteurization

**Figure 6:**
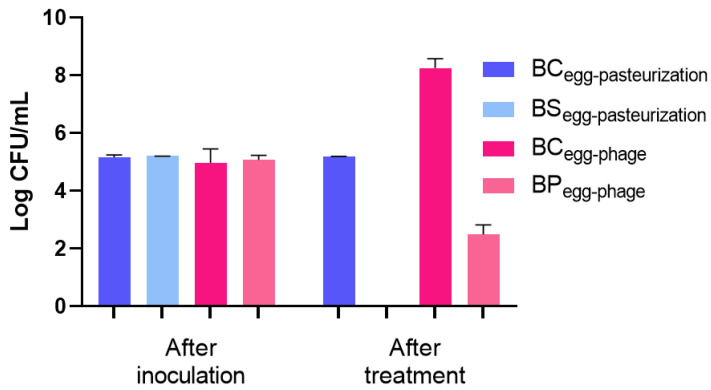
Inactivation of *S.* Enteritidis in liquid whole eggs by pasteurization (60 °C for 3.5 min) and by phage PCSE1 treatment (MOI 1000 after 12 h). BC_egg-pasteurization_—bacterial control for pasteurization with only bacterium, maintained at room temperature for 3.5 min (same duration as the heat treatment); BS_egg-pasteurization_—bacterial sample subjected to pasteurization (60 °C for 3.5 min); BC_egg-phage_—bacterial control for phage treatment with only bacterium, maintained at 25 °C for 12 h (same duration as the phage assay); BP_egg-phage_—bacterial sample treated with phage PCSE1 at MOI 1000 for 12 h at 25 °C. After inoculation refers to the time immediately following artificial contamination of the liquid egg in all conditions. Values represent the mean of three independent tests, with error bars representing the standard deviation. Values shown as 0 Log CFU/mL correspond to counts below the detection limit (1 CFU/mL).

A decrease in *S.* Enteritidis concentration to the detection limit of the method with a 5.2 log CFU/mL reduction was observed in the heat-treated samples (BS_egg-pasteurization_) compared to the untreated bacterial control (BC_egg-pasteurization_) (*p* < 0.05, Figure 6). This reduction was comparable to that obtained by the phage PCSE1 treatment (BP_egg-phage_) at MOI 1000 after 12 h (5.8 log CFU/mL), relative to the BC_egg-phage_ control, as shown in Figure 6. No bacteria were detected in the liquid whole egg control (not artificially contaminated and untreated).

### 2.4. Effect of Phage and Pasteurization on Liquid Whole Egg Properties

#### 2.4.1. Physicochemical Analyses

##### pH and Color

The pH values measured in this study ranged from 8.04 (non-treated liquid whole eggs) to 8.06 and 8.10 for treated samples with phage and pasteurization, respectively (*p* > 0.05, Table 1). Regarding the color, although the lowest values were observed in pasteurized eggs, in general, all treated samples showed a similar lightness (L*), redness (a*), and yellowness (b*) (*p* > 0.05) to non-treated eggs. However, a significantly higher color variation (ΔE*) was obtained in the eggs treated with pasteurization (ΔE* = 5.04) compared to eggs treated with the phage (ΔE* = 2.96) (Table 1).

##### Soluble Protein

Liquid whole eggs treated with the phage and pasteurization presented a similar (*p* > 0.05) soluble protein content (27.22 ± 0.38 and 25.76 ± 2.92 mg/mL, respectively) to that obtained in non-treated liquid whole eggs (24.48 ± 2.37 mg/mL) (Table 1).

##### Apparent Viscosity

The apparent viscosity of non-treated liquid whole eggs was 13.58 ± 1.1 mPa·s (Table 1). Similar results (*p* > 0.05) were obtained for liquid whole eggs treated with the phage (13.57 ± 1.0 mPa·s), contrary to the results obtained for eggs treated by pasteurization (53.03 ± 15.44 mPa·s) with a significantly higher apparent viscosity (*p* < 0.05).

#### 2.4.2. Functional Analyses—Foaming Properties

The influence of the studied treatments on the foaming properties of liquid whole eggs are presented in Table 1. Despite the lower foaming capacity observed for liquid whole eggs treated with pasteurization, no significant differences were observed between the treated samples and non-treated liquid whole eggs (*p* > 0.05). Regarding foaming stability, the lowest value was obtained for pasteurized eggs, being significantly different to that obtained in phage-treated eggs (*p* < 0.05). Untreated and phage-treated eggs presented similar foaming stability (*p* > 0.05).

## 3. Discussion

Considering the association between egg ingestion and *Salmonella* infection, particularly by *S.* Enteritidis [7,8], in this study, the potential of phage PCSE1 as a biocontrol agent against *S.* Enteritidis in liquid whole eggs was evaluated and compared to that of conventional thermal pasteurization. The effect of this phage and pasteurization on egg properties was also evaluated to assess phage potential as a strategy to decontaminate liquid egg products.

The evaluation of phage stability under different environmental conditions is an important aspect to consider for phages that are intended to be applied against foodborne bacteria, mainly pH and temperature, because these factors affect the effectiveness of treatments [21,47]. The optimal pH value for high phage activity is between pH 7 and 10, with most phages being susceptible at pH < 4 [48]. Low pH decreases phage stability by promoting the coagulation and precipitation of phage proteins [49]. High temperatures can extend the phage latent period [50] and lead to denaturation of capsid proteins and DNA melting [51]. Considering the pH of liquid whole eggs (approximately 8) [52] and frequent egg storage at 4 and 25 °C, the high stability of phage PCSE1 in these conditions indicates that this phage can be used to control egg contamination by *S.* Enteritidis during long storage periods. Furthermore, the stability of this phage in other conditions, for a long time, even at pH 3, highlights its potential applicability in other foods, including acidic ones. Phage PCSE1 titer remained stable at pH 7 and 8, with a slight decrease at pH 5 after 56 days and a decrease to the detection limit at pH 3 only after 10 days, being also stable at 4, 25, and 37 °C, with only a slight decrease at 45 °C after 56 days.

Previous studies have also evaluated the stability of *S.* Enteritidis phages under different pH and temperature conditions, although the evaluation was performed mostly over short storage periods [40,41,42]. Phage STG2 remained stable at pH 6–9 but showed a 6 log PFU/mL reduction at pH 3 and dropped to the detection limit at pH 2 after 1 h at 37 °C. This phage was also stable at 40–60 °C for 30 min [40]. Similarly, phage EP01 was stable at pH 5–10 but lost viability at pH 3 after 2 h at 37 °C. This phage was also stable at 30–60 °C for 1 h [41]. Phage vB_SenP_P32 maintained stability across pH 2–12 after 24 h at 25 °C, with reductions of approximately 10, 25, and 50% at 25, 37, and 45 °C, respectively, after 1 h [42]. Phage vB_SenM_P7 showed reduced stability at pH ≤ 7, with a 25% titer decrease at pH 2 after 24 h at 25 °C [42].

Before phage application to food, it is important to study the impact of MOI on phage efficacy in liquid culture media, to serve as a reference for selecting the best conditions for food testing. In TSB at 25 °C, the maximum *S.* Enteritidis difference relative to the control was similar for the different MOIs tested (10, 100, and 1000). Although an increase in MOI did not result in higher differences, an earlier effect was seen for the highest MOI of 1000 compared to the other tested MOIs (10 and 100). Similar results were also obtained by Brenner et al. (2024) [39], who observed that a higher MOI resulted in the faster prevention of bacterial growth in TSB. Phage PCSE1 efficacy in TSB was also temperature-dependent. At 10 °C and MOI 100, although a similar bacterial reduction was obtained to that at 25 °C, the 10 °C reduction took longer. At 4 °C, the maximum prevention of bacterial growth was only obtained at the end of treatment. Low temperatures may reduce phage efficacy and extend the time required for bacterial lysis due to the decreased metabolic activity of the host bacteria. Similar results were also observed by Guo et al. (2021), who reported a slightly stronger antibacterial efficacy of phages against *Salmonella* spp. at 25 °C compared to 4 °C for different tested matrices [53]. In another study, phage EscoHU1 showed a higher and faster prevention of *Salmonella* growth at 25 °C compared to 4 °C at an MOI of 10.000 [54].

Regarding phage replication, an MOI-dependent replication was observed at 25 °C, with a higher replication for lower MOI values. In general, at higher MOIs, low or no phage replication occurs, and lysis is often due to lysis from without [55]. However, in this study, phage replication was seen even at a high MOI of 1000. At refrigeration temperatures and an MOI of 100, phage replication was temperature-dependent with an increase in phage titer at 10 °C but no phage replication at 4 °C. These results are probably due to the non-optimal growth of the bacterial host at 4 °C compared to 10 °C. Most *Salmonella* grows at a temperature range of 7–48 °C, with an optimum around 37 °C [56]. Thus, low temperatures can affect phage replication and, consequently, its efficacy, since phage replication depends on a symbiotic relationship between phages and their bacterial hosts [33].

In liquid whole eggs, at 25 °C, phage efficacy was MOI-dependent and more effective at the highest MOI (1000). However, a higher increase in phage PCSE1 titer was observed for lower values of MOI. Similar results were also obtained by Azari et al. (2023) [23] in liquid whole eggs at 25 °C, with a higher reduction at an MOI of 10,000 compared to an MOI of 10.

In general, bacterial lysis is hampered in food matrices compared to culture media due to the higher complexity and organic matter content in food matrices that can impact phage efficacy by lowering the probability of phages and bacteria interacting. The food itself may include compounds that inhibit or boost phage lytic activity [23]. Furthermore, compared to other liquid foods, liquid eggs may significantly hinder the antibacterial activity of phages [57,58,59], probably due to their high viscosity, which may reduce phage distribution [59]. A reduction in phage titer can actually occur in liquid eggs during storage [59]. In this study, for the same value of MOI 1000, a higher bacterial difference in samples, relative to the bacterial control, was obtained in liquid whole eggs compared to that obtained in the liquid medium TSB. This can be due to the presence of antimicrobial compounds in eggs such as lysozyme, ovalbumin, and ovotransferrin present in egg whites [60]. Although the egg white is the main line of defense against invading microorganisms, several egg yolk components have also demonstrated antimicrobial activity, such as immunoglobulin Y, lipids, and phosvitin [60]. These different antimicrobial substances may act together and have a combined effect with phages. However, these antimicrobial substances present in eggs do not appear to significantly affect phage stability, since phage PCSE1 remained stable in the phage controls in liquid whole eggs, during all the incubation periods for the different tested MOIs.

In TSB, although, in general, a higher bacterial difference in samples relative to the bacterial control was obtained at 25 °C, this temperature was also associated with higher bacterial regrowth compared to that observed at 10 °C and no regrowth at 4 °C. At 25 °C, bacterial regrowth was delayed in liquid whole eggs compared to that observed in TSB. This may be due to the antibacterial substances present in eggs which, in addition to acting in combination with phages improving antibacterial activity, can also have an effect of delaying bacterial regrowth. The combination of the phage treatment with other antibacterial approaches, in this case antibacterial compounds present in eggs, can be an effective solution by decreasing the probability of a bacterial population evolving and becoming resistant to both approaches. Bacteria may become resistant to one approach but not to the others [61]. The level of *Salmonella* contamination applied in this study can also affect observed bacterial regrowth. In practice, the level of *Salmonella* contamination is typically lower than that used in laboratory experiments. Thus, although significant *Salmonella* growth prevention via phage has been observed in eggs, even better than in TSB, these results can probably be improved in real-case scenarios [28].

Regarding the bactericidal effect of thermal pasteurization against *S.* Enteritidis and the maximum bacterial difference obtained with phage PCSE1, relative to the control, they are consistent with the criteria established by the FDA that pasteurization or any other treatment that claims to inactivate *S.* Enteritidis in shell eggs or egg products must achieve a minimum reduction in this pathogen of 5 log [62]. Other studies have also reported similar results for the same conditions of thermal pasteurization used in this study (60 °C, 3.5 min) against *Salmonella* in liquid whole eggs [12,63].

Phage PCSE1 was better at preserving egg properties compared to pasteurization. Considering color variation, this was significantly different between the two treatments. The higher color variation (ΔE*) obtained after treatment with pasteurization compared to that obtained with the phage could indicate a lower presence of carotenoids [64]. This variation was detected by the naked eye (ΔE* > 3) [65] contrarily to that observed for liquid whole eggs treated with phage (ΔE* < 3).

A decrease in soluble protein content typically indicates a reduction in the functionality of egg proteins [64]. Heat can cause egg proteins to unfold, exposing buried hydrophobic groups. This exposure promotes aggregation through hydrophobic interactions, reducing the soluble protein content [66]. However, in this study, both untreated and treated eggs (pasteurization or phage) presented a similar soluble protein content. This is probably due to a moderate denaturation of the proteins associated with the temperature of 60 °C used in this study. Despite the soluble protein data not showing a significant difference between treatments, the higher impact on viscosity caused by pasteurization may indicate some protein unfolding and the consequent aggregation of unfolded proteins under these conditions due to greater protein–protein interactions [67]. Other studies have also observed an increase in viscosity after the pasteurization of liquid whole eggs at 60 °C for 3.5 min [63,64].

Lately, the foaming properties of liquid whole eggs are primarily due to the proteins in egg whites, while the egg yolk acts as an inhibitor because its compounds (proteins and lipids) compete with the egg white proteins [68]. The results of foaming capacity revealed no differences between the treated samples and untreated eggs. However, a considerable decrease in foaming stability was observed for liquid whole eggs treated with pasteurization compared to untreated and phage-treated eggs, probably due to the heat-induced denaturation of egg white proteins, which impairs their ability to stabilize air bubbles. Similar reductions in foaming stability following thermal treatment have been reported in previous studies on liquid whole eggs [64,69].

The results of this study highlight the use of phage PCSE1 in combination with the antibacterial substances present in eggs as a potential strategy to fight *S.* Enteritidis contamination in liquid egg products, preserving at the same time egg properties. In the future, it will be important to evaluate the shelf life of eggs treated with phage PCSE1. Also, it will be important to evaluate the potential of this phage in terms of bacterial lysis and regrowth in liquid whole eggs at refrigeration temperatures. The effectiveness of this phage at 25 °C suggests that the storage of liquid eggs at room temperature may be possible, leading to energy savings.

Although no genes codifying to toxins, antibiotic resistance and integrase enzymes were identified in the phage PCSE1 genome, indicating its potential safety as a biocontrol agent against *S.* Enteritidis, this phage was only partially purified with no remotion of potential bacterial endotoxins. Endotoxin removal is however a costly process, and therefore it is only worthwhile after proving a phage’s potential for application in food matrices, as has been achieved in this study. Thus, in the future, it is necessary to remove the bacterial endotoxins and other potential toxic components for the safe application of this phage in food for human consumption.

## 4. Material and Methods

### 4.1. Bacterium and Culture Conditions

The *S.* Enteritidis (CVB) used in this work is a food isolate gently provided by Controlvet Laboratory [70]. Firstly, this bacterium was grown in Tryptic Soy Agar (TSA, Liofilchem, Roseto degli Abruzzi, Italy) at 37 °C for 24 h and posteriorly kept at 4 °C. Before each test, two colonies were aseptically transferred to 30 mL of Tryptic Soy Broth (TSB, Liofilchem) and incubated overnight at 37 °C, with continuous shaking (120 rpm) until reaching an optical density at 600 nm (OD600) of 0.8, which corresponds to ≈10^9^ colony-forming units (CFU)/mL.

### 4.2. Phage Stock

The phage used against *S.* Enteritidis was phage vB_SeEM_UALMA_PCSE1 (PCSE1), previously isolated from the sewage network of Aveiro (station EEIS9 of SIMRIA Multi Sanitation System of Ria de Aveiro) [46]. Phage PCSE1 (GenBank: PQ314491) belongs to the Caudoviricetes Class and exhibits a myovirus morphotype characterized by an icosahedral head measuring about 71.47 ± 4.55 nm in diameter and a long, retractable tail approximately 121.88 ± 3.40 nm in length, producing small lysis plaques [46]. This phage with double-stranded DNA was selected for this study considering its potential safety, without any known genes codifying to toxins, antibiotic resistance, and integrase enzymes, and also its potential to be applied in food contaminated with other bacterial strains. The results of the phage–host range revealed that phage PCSE1, in addition to its host bacterium, produced fully cleared zones on 6 out of the 57 bacterial strains tested. Among these, PCSE1 formed visible lysis plaques specifically on *S. Enteritidis* CVA and *E. coli* ATCC 25922 [46].

The phage PCSE1 stock used in this study was prepared from an existing stock propagated with *S. Enteritidis* CVB as the host. Briefly, 200 µL of an actively growing *S. Enteritidis* culture was mixed with 5 mL of TSB top agar (0.6% agar concentration; consisting of 30 g/L TSB (Liofilchem), 6 g/L agar (Liofilchem), 0.12 g/L MgSO_4_ (Sigma-Aldrich, St. Louis, MO, USA), and 0.05 g/L CaCl_2_ (Sigma-Aldrich), adjusted to pH 7.4) and poured over a TSA plate. Once the agar solidified, 100 µL of the phage stock was applied beneath the agar layer, and plates were incubated at 25 °C for a minimum of 12 h. This overlay procedure was repeated several times to increase phage concentration. Following incubation, the agar layers were scraped into a flask containing Saline Magnesium (SM) buffer (8 mM MgSO_4_ (Sigma-Aldrich, St. Louis, MO, USA), 0.1 M NaCl (Sigma-Aldrich), 20 mM Tris-HCl (Sigma-Aldrich), pH 7.5) and incubated at 25 °C with gentle shaking at 60 rpm for at least 24 h. The mixture was then centrifuged at 10,000× *g* for 10 min, and the resulting supernatant was filtered through a 0.22-μm membrane filter (Whatman^TM^ GE Healthcare Life Science, Little Marlow, Buckinghamshire, UK) to remove bacterial cells and debris. The phage titer of the newly prepared stock was calculated using the double-layer agar and drop plate methods [71,72]. Serial dilutions of the phage suspension were prepared in Phosphate Buffered Saline (PBS—137 mmol^−1^ NaCl (Sigma-Aldrich), 2.7 mmol^−1^ KCl (Sigma-Aldrich), 8.1 mmol^−1^ Na_2_HPO_4_·2H_2_O, 1.76 mmol^−1^ KH_2_PO_4_ (Sigma-Aldrich), pH 7.4). Two drops of 5 µL from each dilution were plated on TSA plates overlaid with 5 mL of 0.6% TSB top agar containing 200 µL of grown *S. Enteritidis*. Plates were incubated at 25 °C and checked for lytic plaques after 12 h. Plaque counts were used to calculate phage concentration and expressed as plaque-forming units (PFU) per milliliter. Phage PCSE1 stock was kept at 4 °C in SM buffer for several months without significant loss of titer.

### 4.3. Phage Stability Under Different Temperature and pH Conditions

Prior to lytic activity evaluation in TSB and food, phage PCSE1 stability was first assessed in PBS under different values of temperature and pH (initial phage titer ≈ 10^7^ PFU/mL), since phage stability in food may be affected by different conditions of temperature and pH.

To measure the impact of temperature on phage stability, phage suspensions were stored at a certain temperature (4, 25, 37, and 45 °C) and pH 7. Aliquots were collected at 0, 7, 14, 28, 42, and 56 days.

To study the influence of pH on phage stability, phage suspensions at different values of pH (3, 5, 7, and 8) were kept at 25 °C. In the assays at pH 5, 7, and 8, samples were collected at 0, 7, 14, 28, 42, and 56 days. At pH 3, the samples were collected at 0, 1, and 2 h, and after 1, 8, and 10 days, considering the highest reduction in phage titer at lower pH values [40]. Although the pH of liquid whole eggs is around 8 [52], other pH values were tested considering the results of plating efficiency and the suitability of this phage for use in food applications other than liquid eggs against different strains, namely, against *E. coli*.

The phage titer was assessed following the procedure outlined in Section 4.2, with the method’s detection limit set at 1 PFU/mL. Each condition was tested in three independent experiments.

### 4.4. Bacterial Growth Inhibition Curves with Phage Treatment in TSB

Following stability evaluation, in vitro assays in TSB were conducted to assess the effect of different MOIs and temperatures on phage efficacy against *S.* Enteritidis to thus select the best conditions for food assays. *S.* Enteritidis (≈10^5^ CFU/mL) was tested with phage PCSE1 at MOI 1, 10, 100, and 1000 (≈10^5^, 10^6^, 10^7^, or 10^8^ PFU/mL, respectively) at 25 °C and at MOI 100 for the assays at 4 and 10 °C, without agitation (samples—BP_TSB_), considering the results obtained at 25 °C. For each experiment, controls were prepared: one containing only the bacterial culture (BC_TSB_) and another containing only the phage suspension (PC_TSB_). Both controls were incubated under the same conditions as the experimental samples. After 0, 2, 4, 6, 8, 10, 12, and 24 h, aliquots of the samples and controls were collected for the assays at 25 °C, and additionally after 48 and 72 h, for the assays at 4 and 10 °C, since it is expected that at lower temperatures bacterial lysis takes longer as phage adsorption may be hampered at these temperatures [73]. After successive dilutions in PBS, 10 μL per dilution (in duplicate) was plated on TSA plates, which were then maintained at 37 °C for 18–24 h. The bacterial concentration was calculated by counting the colonies from the dilution that provided the most reliable count, with results in CFU/mL (method detection limit: 1 CFU/mL). Phage titer was quantified as described earlier in Section 4.2. All conditions were tested in triplicate through three independent experiments.

### 4.5. Bacterial Kill Curves in Liquid Whole Eggs

First, fresh intact eggs obtained from a local supermarket were surface-disinfected using 70% ethanol. After breaking the eggs, their contents were transferred into a stomacher bag, homogenized for approximately 1 min, and then distributed into sterile Falcon tubes. Liquid whole eggs were then inoculated with *S.* Enteritidis (≈10^5^ CFU/mL).

#### 4.5.1. Phage Treatment

Phage PCSE1 was added to already inoculated samples with *S.* Enteritidis at an MOI of 1, 10, 100, and 1000 (≈10^5^, 10^6^, 10^7^, and 10^8^ PFU/mL, respectively)—sample (BP_egg_). In all assays, controls consisting of only bacteria (BC_egg_) and only phages (PC_egg_) were included. An additional control, the egg control (C_egg_), without bacteria and phages, was also included to evaluate egg natural contamination. After vortexing to ensure the even mixing of phages and bacteria in the viscous liquid whole eggs, both controls and samples were held at 25 °C without shaking.

This temperature was selected since phage adsorption, and its consequent efficacy, may be hampered at refrigeration temperatures [73], considering the results obtained in TSB at refrigeration temperatures. Aliquots were collected at 0, 4, 8, 12, and 24 h, and also at 48 and 72 h. Phage and bacterial concentrations (method detection limit = 1 PFU/mL or 1 CFU/mL, respectively) were measured as detailed in Section 4.2 and Section 4.4, respectively. Each condition was tested in three independent experiments.

#### 4.5.2. Thermal Pasteurization

Non-inoculated egg controls and inoculated bacterial controls (BC_egg-pasteurization_) and samples (BS_egg-pasteurization_) were aseptically transferred into polyamide-polyethylene bags (Plásticos Macar Lda., Santo Tirso, Portugal) that had been previously sterilized using ultraviolet light. The bags were heat-sealed, ensuring minimal air remained inside. The samples (BS_egg-pasteurization_) were subjected to thermal pasteurization in a circulating water bath (Circulator Bath, FALC, Treviglio, Italy) set at 60 °C, with the temperature monitored by a K-type thermocouple (Thermometer 305, Roline, Bassersdorf, Switzerland), for 3.5 min [6]. The time started to count following a come-up time of 40 s which was confirmed at the geometric center of the bags (5 × 4 × 0.5 cm). During this treatment time, controls were maintained at room temperature. After treatment, aliquots were collected, and bacterial concentrations were assessed as mentioned in Section 4.4 (method detection limit = 1 CFU/mL). Each condition was evaluated in three independent experiments.

### 4.6. Effect of Phage PCSE1 and Pasteurization on Liquid Whole Egg Properties

The liquid whole egg samples treated with phage PCSE1 or pasteurization underwent different analyses to assess the effect of the different treatments on egg properties compared to non-treated liquid whole eggs. Different physicochemical and functional analyses were performed.

#### 4.6.1. Physicochemical Analyses

##### pH

The pH of the homogenized liquid whole egg samples was measured at 25 °C using a calibrated glass electrode (micropH 2000, Crison Instruments, S.A., Barcelona, Spain) immersed directly in the samples. Measurements were conducted in triplicate for each sample, with each analysis performed three times.

##### Color

The color of both treated and non-treated egg samples was evaluated using a Konica Minolta CM 2300d spectrophotometer (Konica Minolta, Osaka, Japan). The measured color parameters were L* (lightness, where 0 is dark and 100 is light), a* (redness, with + indicating red and -indicating green), and b* (yellowness, with + indicating yellow and - indicating blue). All measurements were performed at room temperature using the CIELab color space system. The parameters in CIELab were calculated with the original SpectraMagicTM NX Software (Konica Minolta, Osaka, Japan), following the standards set by the International Commission on Illumination. For the analysis, 5 mL of homogenized sample was placed in the sample holder. The total color difference (ΔE*) between treated and untreated samples was determined using Equation (1) [74]:

ΔE* = [(L* − *L*_0_*)^2^ + (a* − *a*_0_*)^2^ + (b* − *b*_0_*)^2^]^1/2^ (1)

where ΔE* denotes the overall color difference; L* and *L*_0_* are the lightness values of treated and untreated samples, respectively; a* and *a*_0_* are the redness values; and b* and *b*_0_* are the yellowness values.

##### Soluble Protein

Dilutions of the egg samples to 10% (*m*/*v*) using distilled water were prepared and then centrifuged at 10,000× *g* for 15 min at 4 °C (Heraeus Biofuge Stratos, Thermo Electron Corporation, Waltham, MA, USA) [75]. The concentration of soluble protein in the resulting supernatant was measured using a Bradford assay [76]. For the assay, 200 µL of Bradford reagent was mixed with 40 µL of sample and incubated in the dark while shaking at 70 rpm (Orbital shaker PSU-10i, Biosam, Riga, Latvia). Absorbance readings were taken at 595 nm, 20 min after reagent addition, with a Multiskan GO Microplate Spectrophotometer (Thermo Fisher Scientific Inc., Waltham, MA, USA). Bovine serum albumin (Sigma-Aldrich, Lisbon, Portugal) served as the protein standard to generate a calibration curve between 0.15 and 0.5 mg/mL, as commonly used for protein quantification [76]. All samples were measured in triplicate, with each analysis performed twice, and the results were presented in mg/mL.

##### Viscosity

A rotational shear rheometer (Kinexus PRO, Malvern Panalytical, Malvern, UK) with an attached cone-and-plate geometry (stainless steel cone, 4° and diameter of 40 mm) was used to assess viscosity. The temperature was maintained at 20.0 ± 0.1 °C using a Peltier system integrated into the lower plate of the rotational rheometer. Following the transfer of the sample to the rheometer plate, a shear rate ramp was applied for 5 min, gradually increasing from 0.1 to 200 s^−1^. rSpace software (Malvern Panalytical, version 1.76s) was employed to calculate the apparent viscosity and to generate the flow curves. For sample comparison, the apparent viscosity at a shear rate of 57 s^−1^ was considered. All rheological measurements were carried out in triplicate.

#### 4.6.2. Functional Properties Analysis: Foaming Capacity and Stability

Foaming properties analysis was determined following the procedure described by Sheng et al. [75]. To generate the foams, 4.5 mL of each egg sample was whipped for 2.5 min using a laboratory homogenizer (Ultra-Turrax, T25 basic, IKA^®^-Werke, Staufen, Germany) set at 9500 rpm, at ambient temperature, within a 20 mL measuring cylinder. Foaming capacity (*FC*) was determined by calculating the percentage increase in foam volume over the initial liquid volume, calculated according to Equation (2):

(2) $$FC\left(\%\right)=\frac{V_{1}}{V_{0}}\;\times \;100$$

Foam stability (*FS*) was evaluated by measuring the decrease in foam volume after 30 min of rest at room temperature and determined according to Equation (3):

(3) $$FS\left(\%\right)=\frac{V_{2}}{V_{1}}\;\times \;100$$

In these equations, *V*_0_ represents the initial liquid volume (mL), *V*_1_ corresponds to the foam volume immediately after whipping (mL), and *V*_2_ is the foam volume after 30 min (mL). All foaming measurements were performed in triplicate for each sample.

### 4.7. Statistical Analysis

Statistical analysis was conducted using GraphPad Prism version 8.4.3. The normality of the data distribution was verified within the same software. Differences in bacterial and phage concentrations across treatments and over time in the killing curve assays were analyzed by a two-way analysis of variance (ANOVA), followed by a Tukey’s multiple comparison test for pairwise comparisons of the means. For each time point, the results from treated samples were compared with their corresponding controls to assess significant differences. The impact of each treatment on eggs’ properties was evaluated through a one-way ANOVA, also followed by a Tukey’s multiple comparison to determine significant differences between treatments. A *p*-value of less than 0.05 was considered statistically significant. All experiments were independently repeated three times, with two replicates per condition.

## 5. Conclusions and Future Perspectives

Phage PCSE1 was effective against *S.* Enteritidis in liquid whole eggs, delaying bacterial regrowth and showing a similar bacterial reduction to that obtained with conventional pasteurization (5.8 log CFU/mL with the phage against 5.2 log CFU/mL with pasteurization). These results together with phage PCSE1’s stability at pH 7 and 8 and temperatures of 4, 25, and 37 °C during long storage periods (56 days) and the preservation of the original egg properties, when compared to pasteurization, highlight the potential application of phage PCSE1 as a promising strategy to decontaminate liquid egg products. In the future, it is important to evaluate the effectiveness of this phage in controlling different *S*. Enteritidis strains.

Phages represent a promising eco-friendly strategy for improving food safety. Despite growing evidence of their efficacy and the approval of several commercial products, important challenges remain regarding regulatory approval, industrial scalability, and practical implementation. Regulatory frameworks are still inconsistent across regions, requiring clearer guidelines on phage safety, efficacy, and stability. Industrial-scale production must be economically viable and ensure phage activity is preserved during processing, storage, and application. Moreover, consumer acceptance is a significant barrier, highlighting the need for better communication about the safety and natural occurrence of phages. Continued research is needed to address potential phage resistance, assess long-term effects on microbial communities, and optimize application strategies [18].

## Figures and Tables

**Table 1:** Physicochemical and functional properties of untreated and treated liquid whole eggs.

Properties	Untreated Liquid Whole Eggs	Pasteurization	Phage Treatment
pH	8.04 ± 0.16 ^a^	8.10 ± 0.15 ^a^	8.06 ± 0.16 ^a^
L*	46.04 ± 2.90 ^a^	42.84 ± 0.98 ^a^	45.27 ± 0.68 ^a^
a*	11.50 ± 1.06 ^a^	10.31 ± 0.55 ^a^	11.78 ± 0.67 ^a^
b*	24.11 ± 3.25 ^a^	20.55 ± 1.24 ^a^	23.96 ± 2.80 ^a^
∆E*	-	5.04 ± 1.33 ^a^	2.96 ± 0.83 ^b^
Soluble protein (mg/mL)	24.48 ± 2.37 ^a^	25.76 ± 2.92 ^a^	27.22 ± 0.38 ^a^
Viscosity (mPa·s, shear rate 57 s^−1^)	13.58 ± 1.1 ^a^	53.03 ± 15.44 ^b^	13.57 ± 1.0 ^a^
Foaming capacity (%)	155.56 ± 9.07 ^a^	140.74 ± 5.24 ^a^	151.85 ± 5.24 ^a^
Foaming stability (%)	48.89 ± 3.14 ^a^	35.56 ± 3.14 ^b^	44.44 ± 3.14 ^a^

Different letters along each row indicate significant differences (*p* < 0.05) between conditions.

## Data Availability

The original contributions presented in this study are included in the article. Further inquiries can be directed to the corresponding author.
